# Draft genome sequence of *Chryseobacterium cucumeris* WMS2 isolated from the rhizosphere soil of watermelon (*Citrullus lanatus*) in Bangladesh

**DOI:** 10.1128/mra.00686-26

**Published:** 2026-09-21

**Authors:** Rofiqul Islam Nayem, Mst. Raihana Sultana, Yang Zhang, Mst. Mutmainna Mitu, Jannatun Bahar Purba, Md. Mahidul Islam Masum

**Affiliations:** 1 Department of Plant Pathology, Gazipur Agricultural University 198780, Gazipur, Bangladesh; 2 Institute of Biotechnology and Food Science, Hebei Academy of Agricultural and Forestry Sciences, Shijiazhuang, Hebei, China; University of Strathclyde, Glasgow, United Kingdom

**Keywords:** *Chryseobacterium cucumeris*, whole genome, characterization, saline soil

## Abstract

We report the draft genome sequence of *Chryseobacterium cucumeris* strain WMS2, isolated from the salt-affected rhizosphere soil of watermelon at Saint Martin’s Island in Bangladesh. The genome assembly comprised 5,264,214 bp with 36.5% GC content and 4,853 protein-coding genes.

## ANNOUNCEMENT

Microorganisms associated with plant rhizospheres in saline coastal ecosystems play important roles in nutrient cycling and environmental adaptation (1, 2). Members of the genus *Chryseobacterium* are extensively found in soil and aquatic environments (3) and are known for their metabolic versatility (4). However, genome-level characterization of strains from saline coastal agricultural environments remains limited.

In January 2026, strain WMS2 was isolated from rhizosphere soil collected directly from the soil adhering to the roots of a single healthy watermelon (*Citrullus lanatus*) plant in the saline coastal agricultural land of Saint Martin’s Island in Bangladesh (20.6236°N, 92.3227°E). Approximately 1 g of soil was suspended in 9 mL sterile saline solution (0.85% NaCl) and homogenized. The suspension was subjected to 10-fold serial dilutions, and 30 µL aliquots of each dilution were spread onto nutrient agar (NA) (HiMedia, India), followed by incubation at 28°C for 48 h. Selected colonies were repeatedly sub-cultured on fresh NA plates and incubated at 28°C for 24–48 h under the same conditions as the initial isolation to ensure culture purity. Purified isolates were selected based on colony morphology and maintained through repeated streaking. The final pure culture was preserved in 25% (vol/vol) glycerol at −80°C for further analysis and genome sequencing over a period of 2 months. For genomic DNA extraction, a single colony revived from a glycerol stock of a pure bacterial culture on NA plates with a sterile inoculating loop was inoculated into 5 mL of nutrient broth (HiMedia, India) and incubated overnight at 28°C with shaking at 200 rpm. Genomic DNA was isolated with the FavorPrep Tissue Genomic DNA Extraction Mini Kit (FavorGen Biotech Corp., Taiwan) following the manufacturer’s instructions. The purity and concentration of DNA were evaluated utilizing a NanoDrop spectrophotometer (Thermo Fisher Scientific, USA). Draft genome sequencing was performed by an external service provider (Macrogen, Inc., Seoul, Korea) on an Illumina MiSeq platform (600-cycle run). A paired-end Illumina sequencing library (2 × 250 bp) was prepared using the Illumina Nextera XT Kit (5). Library quality and concentration were evaluated using fluorometric quantification and fragment size analysis before sequencing.

A total of 29.37 million paired-end reads were generated, of which 28.92 million high-quality reads were retained after filtering using fastp v1.0.1 (6). *De novo* genome assembly was performed using SPAdes v3.15.5 (7). The genome completeness and contamination were estimated using CheckM v1.2.5 (8), applied to the PGAP gene set with the *Chryseobacterium* CheckM marker set, indicating 99.4% completeness and 3.19% contamination. Default parameters were used for all software unless otherwise specified. The genome annotation was carried out using the NCBI Prokaryotic Genome Annotation Pipeline (v6.11) (9).

The draft genome assembly consists of 284 contigs totaling 5,264,214 bp, with a GC content of 36.5%, an N50 of 497.6 kb, and an L50 of 5 (Table 1). The genome contains 4,963 predicted genes, including 4,853 protein-coding genes and 83 RNA genes (75 tRNAs, 5 rRNAs, and 3 ncRNAs), along with 27 pseudogenes (Table 1). NCBI taxonomy analysis identified the strain as *Chryseobacterium cucumeris* with 98.59% ANI to the type strain genome (GCA_001593385.1).

**TABLE 1:** Genomic features of *Chryseobacterium cucumeris* strain WMS2

Genomic features	Value
Assembly method	SPAdes v3.15.5
Sequencing technology	Illumina MiSeq (2 × 250 bp)
Estimated coverage	~40×
Number of contigs	284
Genome size (bp)	5,264,214
GC content (%)	36.5
Contig N50	497.6 kb
L50	5
Total genes	4,963
Protein-coding genes	4,853
CDS (total)	4,880
Total RNA genes	83
rRNA genes	5 (1 complete 5S, 1 complete 16S, a complete 23S, 2 partial 16S)
tRNA genes	75
ncRNA genes	3
Pseudogenes	27
Genome completeness (%)	99.4
Contamination (%)	3.19
ANI to type strain (%)	98.59
Annotation pipeline	NCBI Prokaryotic Genome Annotation Pipeline v6.11

## Data Availability

The draft genome shotgun project of *Chryseobacterium cucumeris* strain WMS2 has been deposited in DDBJ/ENA/GenBank under BioProject accession PRJNA1456431 and BioSample accession SAMN57405909. The genome assembly is available under accession JBXRFN000000000, and the version described in this study is JBXRFN010000000. The raw sequencing reads have been deposited in the NCBI Sequence Read Archive under the accession number SRR38404212.
